# Taxonomy and Multi-Gene Phylogeny of Poroid *Panellus* (Mycenaceae, Agaricales) With the Description of Five New Species From China

**DOI:** 10.3389/fmicb.2022.928941

**Published:** 2022-07-27

**Authors:** Qiu-Yue Zhang, Hong-Gao Liu, Viktor Papp, Meng Zhou, Fang Wu, Yu-Cheng Dai

**Affiliations:** ^1^School of Ecology and Nature Conservation, Institute of Microbiology, Beijing Forestry University, Beijing, China; ^2^Faculty of Agronomy and Life Sciences, Zhaotong University, Zhaotong, China; ^3^Department of Botany, Hungarian University of Agriculture and Life Sciences, Budapest, Hungary

**Keywords:** Agaricomycetes, Basidiomycota, new taxa, polypore, wood-decaying fungi

## Abstract

*Panellus* is an Agaricales genus with both lamellate and poroid hymenophore. The poroid species are readily overlooked because of their tiny basidiocarps. The Chinese samples of poroid *Panellus* are studied, and five species, namely *Panellus alpinus*, *Panellus crassiporus*, *Panellus longistipitatus*, *Panellus minutissimus*, and *Panellus palmicola* are described as new species based on morphology and molecular phylogenetic analyses inferred from an nrITS dataset and a multi-gene dataset (nrITS + nrLSU + mtSSU + nrSSU + *tef*1). *Panellus alpinus* is characterized by its round to ellipsoid pores measuring 4–6 per mm and oblong ellipsoid basidiospores measuring 4.8–6 μm × 2.8–3.6 μm; *P. crassiporus* differs from other poroid species in the genus by the irregular pores with thick dissepiments and globose basidiospores measuring 8–9.8 μm × 6.9–8 μm; *P. longistipitatus* is distinguished by its long stipes, pyriform cheilocystidia, and broadly ellipsoid to subglobose basidiospores measuring 7–9.8 μm × 5–7 μm; *P. minutissimus* is characterized by its tiny and gelatinous basidiocarps, 5–20 pores per basidiocarp, and ellipsoid basidiospores measuring 6–8 μm × 3.2–4.2 μm; *P. palmicola* is characterized by its round pores measuring 2–4 per mm, the presence of acerose basidioles, and globose basidiospores measuring 7–9.5 μm × 6.2–8.2 μm. An identification key to 20 poroid species of *Panellus* is provided.

## Introduction

The genus *Panellus* P. Karst. (Mycenaceae, Agaricales), typified by *Panellus stipticus* (Bull.) P. Karst., was established by Karsten (1879). It is characterized by gelatinous to soft corky, pileate to stipitate basidiocarps with both lamellate and poroid hymenophore, thick-walled tramal hyphae, dichophysial hyphae, ellipsoid, ovoid to globose basidiospores, and as causing a white rot (Burdsall and Miller, 1975, 1978; Libonati-Barnes and Redhead, 1984; Corner, 1986; Chang, 1996; Zhang and Dai, 2021).

The taxonomic history of *Panellus* is somewhat ambiguous. In previous studies, the hymenophore structure (e.g., lamella and pores) was considered as an important taxonomic feature on generic level. Later, taxonomists found some intermediate species, such as *Panellus pusillus* (Pers. ex Lév.) Burds. and O. K. Mill. and *Panellus intermedius* Corner with poroid to lamellate hymenophore (Singer, 1953; Corner, 1986). Accordingly, Burdsall and Miller (1975) treated *Dictyopanus* Pat., a poroid mushroom and a subgenus of *Panellus*, and all species of *Dictyopanus* transferred to *Panellus*.

*Panellus* has been extensively studied in the Americas and Europe. However, previous studies mainly focused on lamellate species and only a few reports were provided on poroid species (Earle, 1909; Macrae, 1937; Singer, 1962; Miller, 1970). So far, Corner (1986) reported 13 poroid species of *Panellus*, including eight new species. Johnston et al. (2006) transferred *Favolaschia minima* (Jungh.) Kuntze to *Panellus* based on morphological characteristics and molecular analyses. Zhang and Dai (2021) reported two new poroid species of *Panellus* based on morphology and phylogeny. Currently, more than 50 species are accepted in *Panellus* (Wijayawardene et al., 2022), among them 15 have poroid hymenophore.

This study aims to explore the diversity and phylogeny of *Panellus*, and five new poroid species from China are confirmed to be members of the genus. Currently, the main morphological characteristics of 20 poroid species of *Panellus* are outlined, and an identification key to these species of *Panellus* is provided.

## Materials and Methods

### Morphological Studies

The studied specimens are deposited in the herbarium of the Institute of Microbiology, Beijing Forestry University (BJFC). Morphological descriptions are based on field notes and voucher specimens. Microscopic analyses follow Cui et al. (2019) at a magnification of 1,000× using a Nikon Eclipse 80i microscope (Tokyo, Japan) with phase contrast illumination. In the description, the following abbreviations are used: KOH = 5% potassium hydroxide; IKI = Melzer’s reagent, IKI+ = amyloid; CB = Cotton Blue, CB– = acyanophilous in Cotton Blue; L = arithmetic average of basidiospores length, W = arithmetic average of basidiospores width, Q = L/W ratios, (*n* = x/y) = x measurements of basidiospores from y specimens. Color terms are from Royal Botanic Garden Edinburgh (1969) and Petersen (1996).

### Molecular Studies and Phylogenetic Analysis

A cetyltrimethylammonium bromide (CTAB) rapid plant genome extraction kit (Aidlab Biotechnologies Co., Ltd., Beijing, China) was used to extract DNA (Sun et al., 2020; Liu et al., 2021). The following primer pairs were used to amplify the non-coding genes: ITS5 (5′-GGA AGT AAA AGT CGT AAC AAG G-3′) and ITS4 (5′-TCC TCC GCT TAT TGATAT GC-3′) for the internal transcribed spacer regions (nrITS; White et al., 1990); LR0R (5′-ACC CGC TGA ACT TAA GC-3′) and LR7 (5′-TAC TAC CAC CAA GAT CT-3′) for nuclear large subunit rDNA (nrLSU; Vilgalys and Hester, 1990); MS1 (5′-CAG CAG TCA AGA ATA TTA GTC AAT G-3′) and MS2 (5′-GCG GAT TAT CGA ATT AAA TAA C-3′) for the small subunit mitochondrial rRNA gene (mtSSU); NS1 (5′-GTA GTC ATA TGC TTG TCT C-3′) and NS4 (5′-CTT CCG TCA ATT CCT TTA AG-3′) for the small subunit of nuclear ribosomal RNA gene (nrSSU); as well as protein coding gene: 983F (5′-GCY CCY GGH CAY CGT CAY TTY AT-3′) and 1567R (5′-ACH GTR CCR ATA CCA CCS ATC TT-3′) for translation elongation factor 1α (*tef*1).

The PCR cycling schedules for different DNA sequences of nrITS, nrLSU, mtSSU, nrSSU, and *tef*1 genes used in this study followed those used in Wang et al. (2021) and Zhou et al. (2021). The PCR products were purified and sequenced at the Beijing Genomics Institute (BGI), China with the same primers, and the newly generated sequences were deposited in the GenBank. The sequences generated in this study were aligned with additional sequences downloaded from GenBank (Table 1) using Clustal X (Thompson et al., 1997) and manually adjusted in BioEdit (Hall, 1999).

**TABLE 1:** Taxa information and GenBank accession numbers of sequences used in this study.

Species	Specimen No.	Locality	GenBank accession No.				
			
			nrITS	nrLSU	mtSSU	nrSSU	*tef*1
*Hemimycena mairei*	CBS 263	France	MH856248	MH867779	–	–	–
*H. mairei*	CBS 265	France	MH856249	MH867780	–	–	–
***Panellus alpinus***	Dai 2359**7**	**China**	**ON074661**	**ON074727**	**ON074340**	–	–
***P. alpinus***	Dai 2360**1**	**China**	**ON074662**	–	**ON074341**	**ON074713**	–
*P. bambusicola*	Dai 19899	China	MT363746	–	–	–	–
***P. crassiporus***	Dai 1996**3**	**China**	**ON074663**	**ON074728**	–	**ON074714**	**ON087667**
***P. crassiporus***	Dai 1997**7**	**China**	**ON074664**	**ON074729**	–	**ON074715**	–
***P. crassiporus***	Dai 2366**3**	**China**	**ON074665**	–	–	–	–
***P. crassiporus***	Dai 2366**4**	**China**	**ON074666**	**ON074730**	**ON074342**	**ON074716**	**ON087668**
*P. longistipitatus*	WTUF 043066	United States	MK169353	–	–	–	–
***P. longistipitatus***	Dai 2206**5**	**China**	**ON074667**	**ON074731**	–	**ON074717**	**ON087669**
***P. longistipitatus***	Dai 2248**7**	**China**	**ON074668**	**ON074732**	–	–	–
*P. minimus*	PDD75430	New Zealand	DQ026258	–	–	–	–
***P. minutissimus***	Dai 2205**2**	**China**	**ON074669**	**ON074733**	**ON074343**	**ON074718**	**ON087670**
***P. minutissimus***	Dai 2206**8**	**China**	**ON074670**	**ON074734**	**ON074344**	**ON074719**	**ON087671**
***P. palmicola***	Dai 1970**7**	**China**	**ON074671**	**ON074735**	**ON074345**	**ON074720**	–
***P. palmicola***	Dai 1971**9**	**China**	**ON074672**	**ON074736**	**ON074346**	**ON074721**	**ON087672**
***P. palmicola***	Dai 2232**9**	**China**	**ON074673**	**ON074737**	**ON074347**	**ON074722**	**ON087673**
***P. palmicola***	Dai 2233**0**	**China**	**ON074674**	–	**ON074348**	**ON074723**	**ON087674**
***P. palmicola***	Dai 2233**1**	**China**	**ON074675**	**ON074740**	**ON074349**	**ON074724**	**ON087675**
***P. palmicola***	Dai 2233**2**	**China**	**ON074676**	–	**ON074350**	–	–
***P. palmicola***	Dai 2233**3**	**China**	**ON074677**	**ON074738**	**ON074351**	**ON074725**	**ON087676**
***P. palmicola***	Dai 2233**4**	**China**	**ON074678**	**ON074739**	**ON074352**	**ON074726**	**ON087677**
*P. pusillus*	Dai 20433	China	MZ801774	MZ914394	–	–	–
*P. pusillus*	Dai 20434	China	MZ801775	–	–	–	–
*P. ringens*	MQ18R309	Canada	MN992238	–	–	–	–
*P. ringens*	P. Zhang214	China	KY820048	–	–	–	–
*P. stipticus*	TN 6157	United States	AF289071	–	–	–	–
*P. stipticus*	AODJ. 161.148	–	MZ374496	–	–	–	–
*P. stipticus*	AODJ. 161.146	–	MZ374494	–	–	–	–
*P. stipticus*	TN 9525	Canada	AF289070	–	–	–	–
*P. stipticus*	JP 2998	Finland	MT705625	–	–	–	–
*P. stipticus*	OM14173	Finland	MT705626	–	–	–	–
*P. yunnanensis*	Dai 20728	China	MT300504	MT300511	–	–	–
*P. yunnanensis*	Dai 20729	China	MT300505	MT300512	–	–	–
*P. yunnanensis*	Dai 20730	China	MT300506	MT300513	–	–	–

*New sequences are shown in bold.*

In this study, nuclear ribosomal RNA genes were used to determine the phylogenetic positions of new species. The sequence alignment was deposited at TreeBase (submission ID 29605). Sequences of *Hemimycena mairei* (E-J. Gilbert) Singer were used as outgroup (Vu et al., 2018).

Maximum parsimony (MP), maximum likelihood (ML), and Bayesian inference (BI) were employed to perform the phylogenetic analysis of the two aligned datasets. MP topology and bootstrap (BT) values obtained from 1,000 replicates were computed in PAUP* version 4.0b10 (Swofford, 2002). All characters were equally weighted, and the gaps were treated as missing data. Trees were inferred using the heuristic search option with tree-bisection reconnection (TBR) branch swapping and 1,000 random sequence additions. Max-trees were set to 5000, branches of zero length were collapsed, and all parsimonious trees were saved. Clade robustness was assessed by a BT analysis with 1,000 replicates (Felsenstein, 1985). Descriptive tree statistics, such as tree length (TL), consistency index (CI), retention index (RI), rescaled consistency index (RC), and homoplasy index (HI) were calculated for each maximum parsimonious tree (MPT) generated.

RAxML 7.2.8 was used to construct ML trees for both datasets with the GTR + I + G model of site substitution, including the estimation of gamma-distributed rate heterogeneity and a proportion of invariant sites (Stamatakis, 2006). The branch support was evaluated with a bootstrapping method of 1,000 replicates (Hillis and Bull, 1993).

The BI was conducted with MrBayes 3.2.6 in two independent runs, each of which had four chains for 10 million generations and started from random trees (Ronquist and Huelsenbeck, 2003). Trees were sampled every 1000th generation. The first 25% of the sampled trees were discarded as burn-in and the remaining ones were used to reconstruct a majority rule consensus and calculate Bayesian posterior probabilities (BPPs) of the clades.

Phylogenetic trees were visualized using Treeview (Page, 1996). Branches that received BT support for MP (≥75%), ML (≥75%), and BPPs (≥0.95) were considered as significantly supported.

## Results

### Phylogeny

The nrITS-based phylogeny included nrITS sequences from 38 fungal collections representing 13 species. The dataset had an aligned length of 665 characters, of which 372 characters are constant, 61 are variable and parsimony-uninformative, and 232 are parsimony-informative. MP analysis yielded two trees (TL = 546, CI = 0.771, RI = 0.921, RC = 0.710, HI = 0.229). The best model for the nrITS sequences dataset estimated and applied in the BI was GTR + I + G. BI resulted in a similar topology with an average standard deviation of split frequencies = 0.007304 to MP analysis, and thus only the MP tree is provided.

**FIGURE 1:**
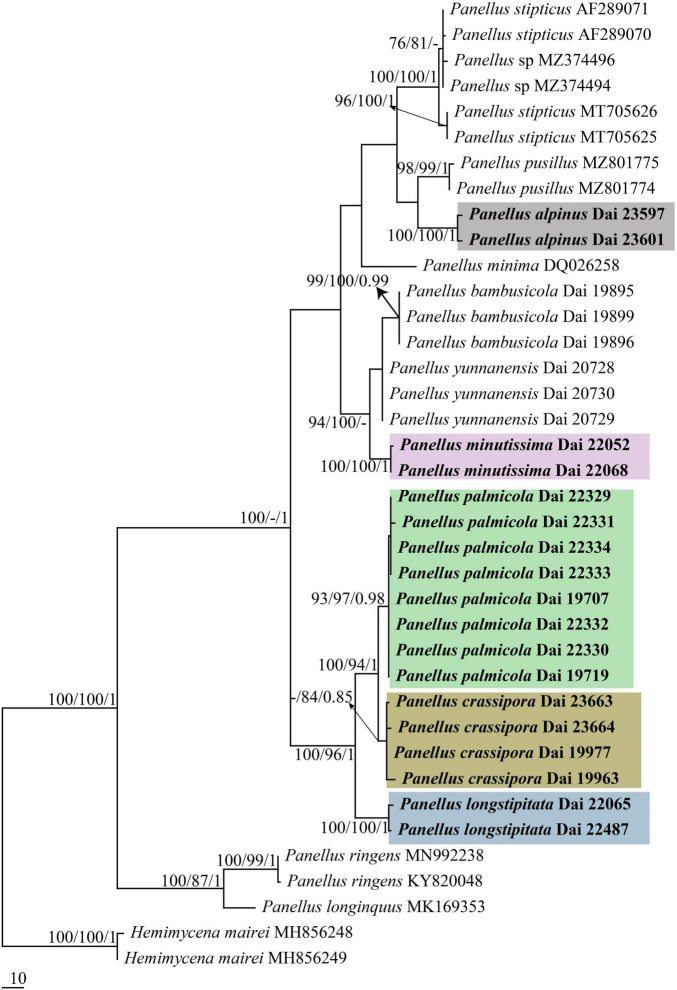
Maximum parsimony (MP) tree illustrating the phylogeny of *Panellus* based on nrITS dataset. Branches are labeled with parsimony bootstrap values (MP/ML) higher than 75%, and Bayesian posterior probabilities (BPPs) more than 0.95. The five colors represent the five new species in this paper, respectively.

**FIGURE 2:**
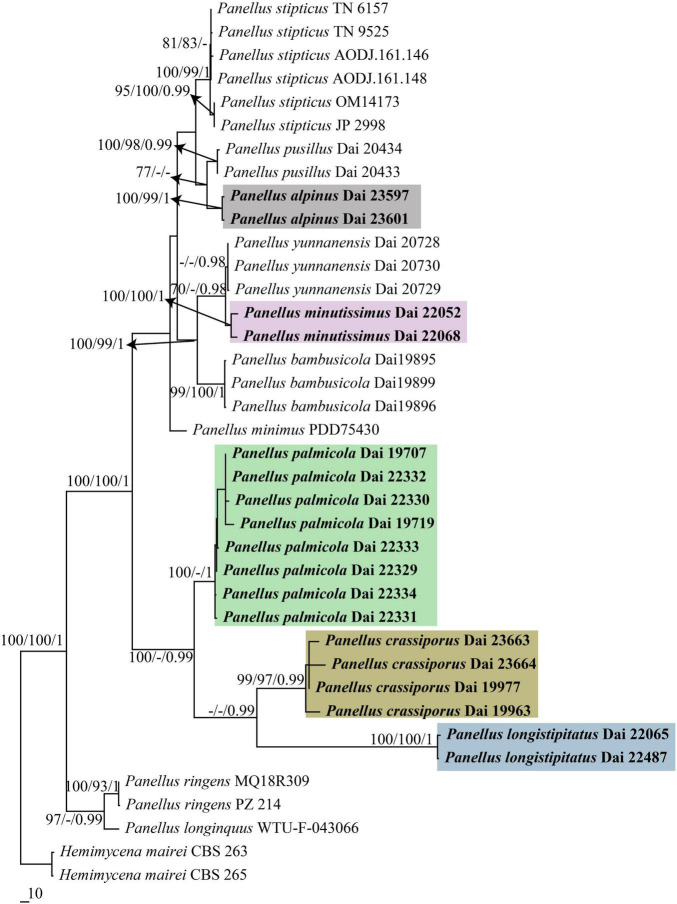
Maximum parsimony (MP) tree illustrating the phylogeny of *Panellus* based on combined 5-gene (nrITS + nrLSU + mtSSU + nrSSU + *tef*1) dataset. Branches are labeled with parsimony bootstrap values (MP/ML) higher than 75% and Bayesian posterior probabilities (BPPs) more than 0.95. The five colors represent the five new species in this paper, respectively.

The combined 5-gene (nrITS + nrLSU + mtSSU + nrSSU + *tef*1) sequence dataset from 38 fungal specimens representing 13 taxa did not show any conflicts in tree topology for the reciprocal bootstrap trees, which allowed us to combine them (*p* > 0.01). The dataset had an aligned length of 3,682 characters, of which 2,932 characters are constant, 266 are variable and parsimony-uninformative, and 484 are parsimony-informative. MP analysis yielded three trees (TL = 1120, CI = 0.846, RI = 0.918, RC = 0.777, HI = 0.154). The best model for the combined nrITS + nrLSU + mtSSU + nrSSU + *tef*1 dataset estimated and applied in the Bayesian analysis was GTR + I + G. Bayesian analysis resulted in a similar topology with an average standard deviation of split frequencies = 0.004519 to MP analysis, and thus only the MP tree is provided. Both BT values (≥75%) and BPP values (≥0.95) are shown at the nodes (Figures 1, 2).

In both nrITS and nrITS + nrLSU + mtSSU + nrSSU + *tef*1 based phylogenies (Figures 1, 2), five new well-supported lineages were formed. Among them, two specimens (Dai 23597 and Dai 23601) from Tibet formed a well-supported lineage (100/100/1 in Figure 1, 100/99/1 in Figure 2), named *Panellus alpinus*, sister to *P. pusillus*. Two specimens (Dai 22052 and Dai 22068) formed a well-supported lineage, named *P. minutissimus* (100/100/1 in Figures 1, 2), and is closely related to *Panellus bambusicola* Q. Y. Zhang and Y. C. Dai and *Panellus yunnanensis* Q. Y. Zhang and Y. C. Dai. In addition, another 14 samples formed three distinct lineages nested in *Panellus*, and they are named *P. crassiporus*, *P. longistipitatus*, and *P. palmicola*, respectively.

### Taxonomy

**FIGURE 3:**
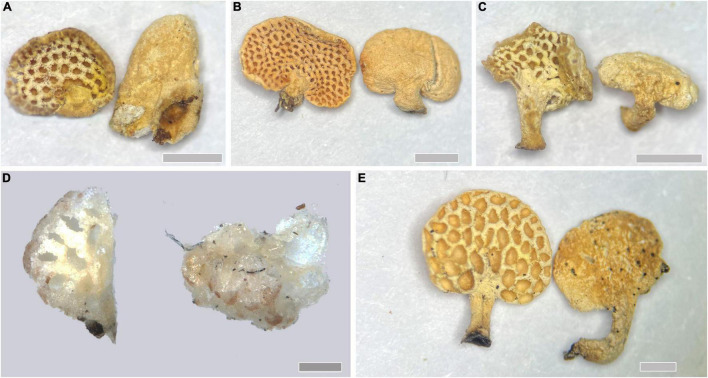
Basidiocarps of *Panellus* species. **(A)** *P. alpinus*; **(B)** *P. crassiporus*; **(C)** *P. longistipitatus*; **(D)** *P. minutissimus*; **(E)** *P. palmicola* [Scale bars: **(A–C,E)** = 1 mm; **(D)** = 0.2 mm].

**FIGURE 4:**
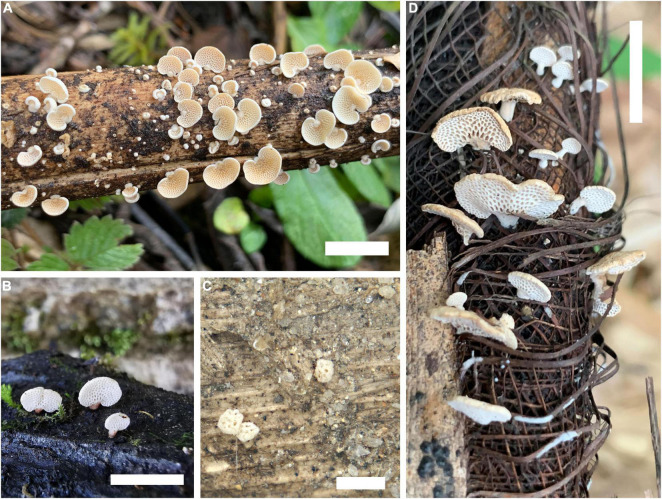
*In situ* photo documentations of *Panellus* species. **(A)** *P. alpinus* (holotype); **(B)** *P. crassiporus* (Dai 23663); **(C)** *P. minutissimus* (holotype); **(D)** *P. palmicola* (Dai 22334) [Scale bars: **(A,B,D)** = 5 mm; **(C)** = 1 mm].

**FIGURE 5:**
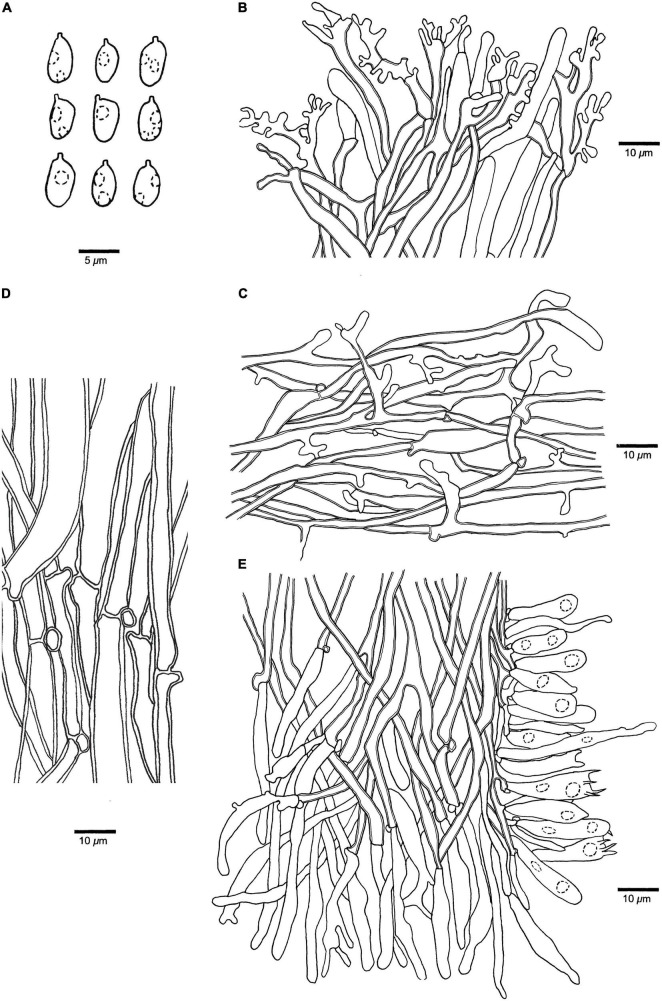
Microscopic structures of *Panellus alpinus* (holotype). **(A)** Basidiospores. **(B)** Dichophysial hyphae and pileocystidia from pileipellis. **(C)** Hyphae from pileus. **(D)** Hyphae from stipe base. **(E)** A section of tube trama, including pleurocystidia, cheilocystidia, basidia, and basidioles. The dotted circles represent some guttules in basidiospores, basidia or basidioles.

***Panellus alpinus*** Q. Y. Zhang, F. Wu, and Y. C. Dai, sp. nov., Figures 3A, 4A, 5.

MycoBank: MB844364.

**Type.** China, Tibet Autonomous Region, Nyingchi, Bomi County, dead bamboo (Bambusoideae), 26 October 2021, Dai 23600 (holotype, BJFC 038172).

**Etymology.** *Alpinus* (Lat.): Referring to the species being found in high-altitude areas.

**Basidiocarps** annual, pileate with a lateral rudimentary stipe base, gregarious, soft corky when fresh, and chalky when dry. **Pileus** 1.5–4 mm × 1–3 mm, flabelliform, semicircular or ellipsoid; pileal surface white to cream when fresh and dry, opaque, convex to plane, pruinose; margin incurved, entire; context thin. **Hymenophore** concolorous with pileal surface, poroid, about 80–150 pores per basidiocarp; mature pores 4–6 per mm, round or elongated to ellipsoid; tubes up to 0.2 mm long. **Stipe base** 0.4–1 mm × 0.2–0.6 mm, concolorous with pileal surface, tapering to the slightly swollen base, and pruinose on surface.

**Basidiospores** (4.5–)4.8–6(–6.5) μm × (2.7–)2.8–3.6 μm, *L* = 5.29 μm, *W* = 3.07 μm, *Q* = 1.70–1.76 (*n* = 90/3), oblong ellipsoid, tapering at apiculus, hyaline, thin-walled, smooth, with some guttules, faintly IKI+, CB–. **Basidia** 17–24 μm × 4–6 μm, clavate with some guttules, 4–spored, sterigmata 2–4 μm long; basidioles 16–24 μm × 4–5 μm, clavate to acerose. **Pleurocystidia** 20–36 μm × 3–4 μm, present in hymenium, narrowly clavate or apiculate, thin-walled. **Cheilocystidia** 25–45 μm × 2–5 μm, present at dissepiment edge, subulate to long fusiform, thin-walled, some with diverticulate projections at the apex. **Pileipellis** comprises a palisade of abundant dichophysial hyphae and pileocystidia; dichophysial hyphae slightly thick-walled, 2–4 μm in diameter; pileocystidia 22–47 μm × 3–5 μm, narrowly clavate, thin-walled, some with diverticulate projections, and sometimes indistinguishable from the dichophysial hyphae. **Pileus** hyphae interwoven, with diverticulate projections, slightly thick-walled, 1.5–5 μm in diameter. **Tramal** hyphae interwoven, occasionally branched, slightly thick-walled, 2–4 μm in diameter. **Hyphae in stipe base** subparallel along stipe, some part swollen, slightly thick-walled, 3–12 μm in diameter. Clamp connections are present.

Additional specimens (paratypes) examined: CHINA, Tibet Autonomous Region, Nyingchi, Bomi County, dead bamboo (Bambusoideae), 26 October 2021, Dai 23597 (BJFC 038169), Dai 23598 (BJFC 038170), Dai 23599 (BJFC 038171), Dai 23601 (BJFC 038173), Dai 23602 (BJFC 038174), Dai 23605 (BJFC 038177).

*Note*. *Panellus alpinus* grows on dead bamboo (Bambusoideae), and it was found in Tibet, China at an elevation of 3,000 m around timberline.

**FIGURE 6:**
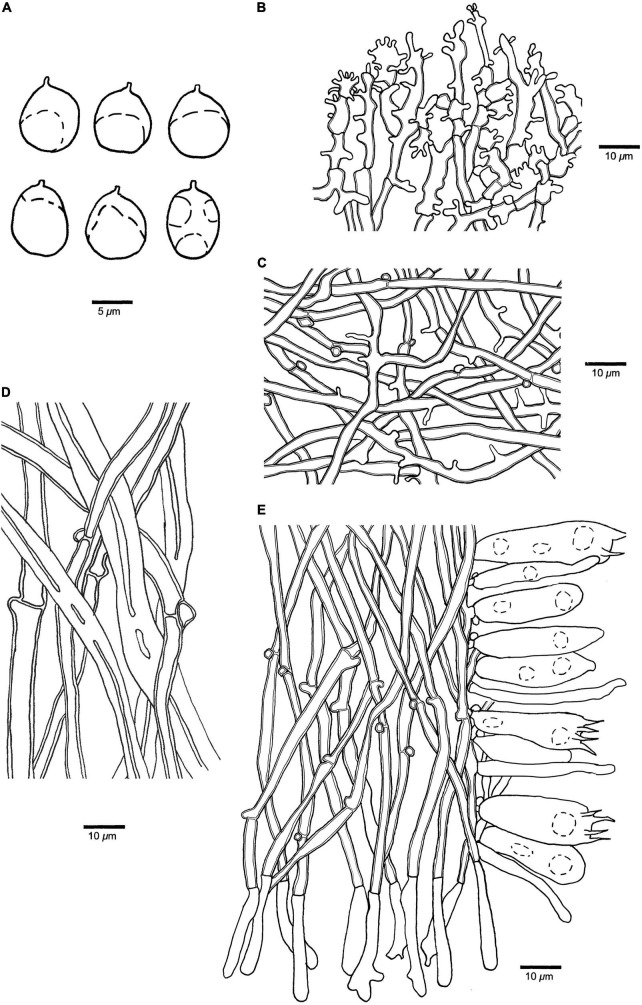
Microscopic structures of *Panellus crassiporus* (holotype). **(A)** Basidiospores. **(B)** Dichophysial hyphae from pileipellis. **(C)** Hyphae from pileus. **(D)** Hyphae from stipe. **(E)** A section of tube trama, including pleurocystidia, cheilocystidia, basidia, and basidioles. The dotted circles represent some guttules in basidiospores, basidia or basidioles.

***Panellus crassiporus*** Q. Y. Zhang, F. Wu, and Y. C. Dai, sp. nov., Figures 3B, 4B, 6.

MycoBank: MB844365.

**Type.** China, Yunnan Province, Wenshan, Xichou County, Xiaoqiaogou Forest Farm, fallen angiosperm trunk, 29 June 2019, Dai 19963 (holotype, BJFC 031637).

**Etymology.** *Crassiporus* (Lat.): referring to the species having thick dissepiments of pores.

**Basidiocarps** annual, gregarious, soft corky when fresh, and chalky when dry. **Pileus** 2–5 × 1–3 mm, reniform or flabelliform; pileal surface white to pale buff when fresh and dry, opaque, convex to plane, pruinose; margin incurved or straight, entire; context thin. **Hymenophore** concolorous with a pileal surface, poroid, about 60–180 pores per basidiocarp; mature pores 4–6 per mm, irregularly angular to round with thick dissepiments; tubes up to 0.4 mm long. **Stipe** 0.1–1.3 mm × 0.1–0.5 mm, concolorous with a pileal surface, laterally attached, short with slightly swollen base, pruinose on the surface.

**Basidiospores** (7.5–)8–9.8(–10) μm × (6.8–)6.9–8(–9) μm, *L* = 8.63 μm, *W* = 7.52 μm, *Q* = 1.13–1.16 (*n* = 90/3), subglobose to globose, hyaline, thin-walled, smooth, with some guttules, faintly IKI+, CB–. **Basidia** 30–36 μm × 8–12 μm, clavate with some guttules, 2(–4)–spored, sterigmata 2–5 μm long; basidioles 22–34 μm × 5–9 μm, clavate, acerose or fusiform. **Pleurocystidia** 18–38 μm × 3–7 μm, present in hymenium, tubular, thin-walled. **Cheilocystidia** 22–30 μm × 3–5 μm, present at dissepiment edge, tubular or narrowly clavate, thin-walled, some with diverticulate projections at the apex. **Pileipellis** comprises abundant, dense dichophysial hyphae; dichophysial hyphae slightly thick-walled, 3–5 μm in diameter; pileocystidia absent. **Pileus hyphae** interwoven, with diverticulate projections, slightly thick-walled, 1.5–5 μm in diameter. **Tramal** hyphae interwoven, slightly thick-walled, 2–4 μm in diameter. **Hyphae in stipe** subparallel along stipe, some part swollen, thick-walled, 3–9 μm in diameter. Clamp connections present.

Additional specimens (paratypes) examined: China, Tibet Autonomous Region, Nyingchi, Medog County, Guoguotang, rotten angiosperm wood, 28 August 2021, Dai 23663 (BJFC 038235), fallen angiosperm trunk, Dai 23664 (BJFC 038236); Yunnan Province, Wenshan, Xichou County, Xiaoqiaogou Forest Farm, rotten angiosperm wood, 29 June 2019, Dai 19977 (BJFC 031651).

*Note*. *Panellus crassiporus* was found in Medog (elevation around 1000 m) of Tibet and Xichou of Yunnan, southwest China, and both areas have subtropical vegetation.

**FIGURE 7:**
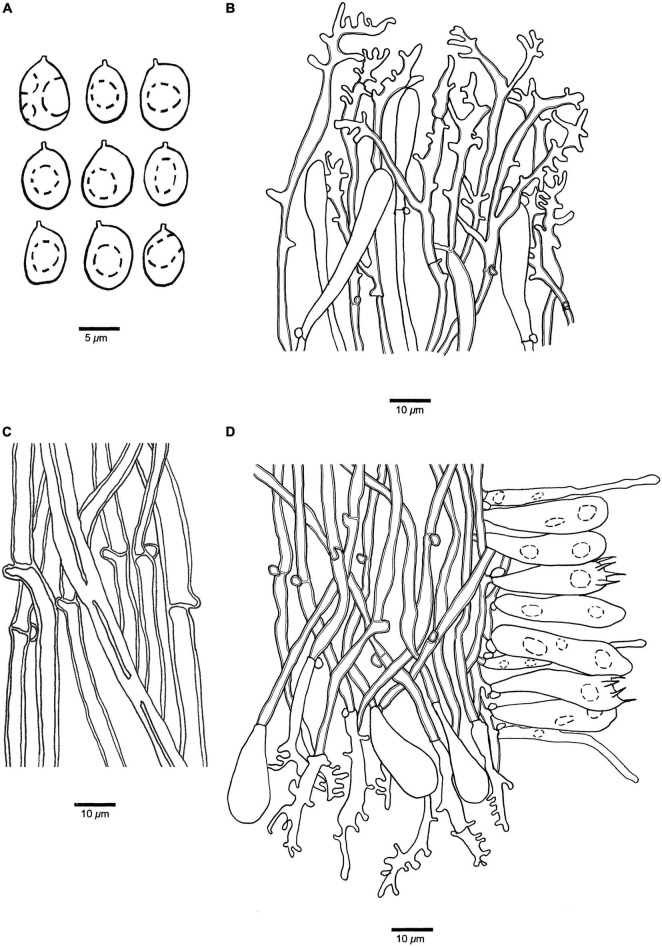
Microscopic structures of *Panellus longistipitatus* (holotype). **(A)** Basidiospores. **(B)** Dichophysial hyphae and pileocystidia from pileipellis. **(C)** Hyphae from stipe. **(D)** A section of tube trama, including pleurocystidia, cheilocystidia, basidia, and basidioles. The dotted circles represent some guttules in basidiospores, basidia or basidioles.

***Panellus longistipitatus*** Q. Y. Zhang, F. Wu, and Y. C. Dai, sp. nov., Figures 3C, 7.

MycoBank: MB844366.

**Type.** Hainan Province, Qiongzhong County, Hainan Tropical Rainforest National Park, Limushan, dead *Trachycarpus fortunei*, 29 June 2021, Dai 22487 (holotype, BJFC 037070).

**Etymology.** *Longistipitatus* (Lat.): referring to the species having a long stipe.

**Basidiocarps** annual, gregarious, soft corky when fresh, and chalky when dry. **Pileus** 1–4.2 × 1–3 mm, reniform to semicircular; pileal surface white to cream when fresh and dry, opaque, convex to plane, pruinose; margin incurved, entire; context thin. **Hymenophore** concolorous with pileal surface, poroid, about 42–164 pores per basidiocarp; mature pores 4–6 per mm, round to irregularly angular; tubes up to 0.3 mm long. **Stipe** 1–3 mm × 0.8–1 mm, concolorous with pileal surface, laterally attached, somewhat reduce to a tapering base, pruinose on surface.

**Basidiospores** 7–9.8(–10) μm × 5–7 μm, *L* = 8.03 μm, *W* = 6.27 μm, *Q* = 1.32–1.33 (*n* = 60/2), broadly ellipsoid to subglobose, hyaline, thin-walled, smooth, with some guttules, faintly IKI+, CB–. **Basidia** 28–36 μm × 5–8 μm, clavate with some guttules, 4–spored, sterigmata 3–6 μm long; basidioles 23–31 μm × 6–9 μm, clavate to acerose. **Pleurocystidia** 35–53 μm × 3–6 μm, present in hymenium, tubular with tapered at the apex, thin-walled. **Cheilocystidia** 15–28 μm × 7–11 μm, present at dissepiment edge, pyriform, thin-walled. **Pileipellis** comprises a palisade of numerous dichophysial hyphae and pileocystidia; dichophysial hyphae slightly thick-walled, 2–5 μm in diameter; pileocystidia 17–40 μm × 5–8 μm, narrowly clavate, thin-walled. **Tramal** hyphae subparallel along tubes, slightly thick-walled, 3–5 μm in diameter; numerous dichophysial hyphae present at dissepiment edge. **Hyphae in stipe** subparallel along stipe, thick-walled, 3–7 μm in diameter. Clamp connections present.

Additional specimen (paratype) examined: China, Hainan Province, Qiongzhong County, Hainan Tropical Rainforest National Park, Limushan, dead *Trachycarpus fortune*, 11 November 2020, Dai 22065 (BJFC 035958).

*Note*. *Panellus longistipitatus* grows on dead palm (*Trachycarpus fortune*) in a tropical forest.

**FIGURE 8:**
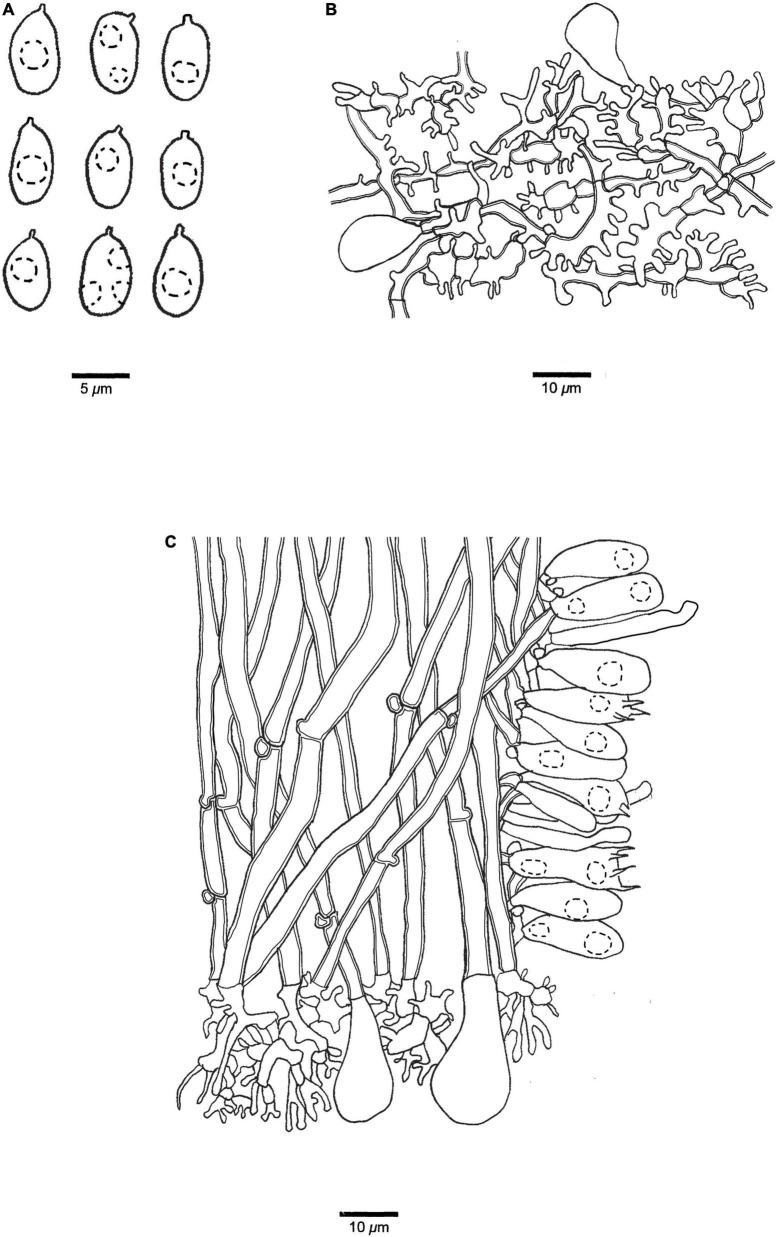
Microscopic structures of *Panellus minutissimus* (holotype). **(A)** Basidiospores. **(B)** Dichophysial hyphae and pileocystidia from pileipellis. **(C)** A section of tube trama, including pleurocystidia, cheilocystidia, basidia, and basidioles. The dotted circles represent some guttules in basidiospores, basidia or basidioles.

***Panellus minutissimus*** Q. Y. Zhang, F. Wu, and Y. C. Dai, sp. nov., Figures 3D, 4C, 8.

MycoBank: MB844367.

**Type.** Hainan Province, Qiongzhong County, Hainan Tropical Rainforest National Park, Limushan, dead bamboo (Bambusoideae), 11 November 2020, Dai 22052 (holotype, BJFC 035946).

**Etymology.** *Minutissimus* (Lat.): referring to the species having tiny basidiocarps.

**Basidiocarps** annual, gregarious, and gelatinous. **Pileus** 0.2–0.6 mm × 0.1–0.6 mm, conchoid, semicircular or ellipsoid; pileal surface pure white to white when fresh, becoming pale cream to ivory upon drying, opaque, convex to plane, slightly powdery appearance; margin incurved; context thin. **Hymenophore** concolorous with pileal surface, poroid, about 4–15 pores per basidiocarp; mature pores 8–10 per mm, round to ellipsoid. **Stipe** absent.

**Basidiospores** 6–8(–8.2) μm × 3.2–4.2(–4.5) μm, *L* = 6.98 μm, *W* = 3.90 μm, *Q* = 1.78–1.79 (*n* = 60/2), ellipsoid to oblong ellipsoid, tapering at apiculus, hyaline, thin-walled, smooth, with some guttules, faintly IKI+, CB–. **Basidia** 18–21 μm × 6–8 μm, clavate with some guttules, 4–spored, sterigmata 1–5 μm long; basidioles 14–20 μm × 4–7 μm, clavate or acerose. **Pleurocystidia** 15–25 μm × 2.5–4 μm, present in hymenium, tubular with slightly curved at the apex, thin-walled. **Cheilocystidia** 20–24 μm × 10–12 μm, present at dissepiment edge, mostly pyriform, thin-walled. **Pileipellis** composed of interwoven dichophysial hyphae and pileocystidia; dichophysial hyphae slightly thick-walled, 2–5 μm in diameter; pileocystidia 12–16 μm × 6–9 μm, pyriform, thin-walled. **Tramal** hyphae subparallel along tubes, slightly thick-walled, 3–6 μm in diameter; numerous dichophysial hyphae present at dissepiment edge, interspersed between cheilocystidia. Clamp connections present.

Additional specimen (paratype) examined: China, Hainan Province, Qiongzhong County, Hainan Tropical Rainforest National Park, Limushan, dead bamboo (Bambusoideae), 11 November 2020, Dai 22068 (BJFC 035961).

*Note*. *Panellus minutissimus* is a gelatinous species with a very small pileus, and it grows on small bamboo (Bambusoideae) in a tropical forest.

**FIGURE 9:**
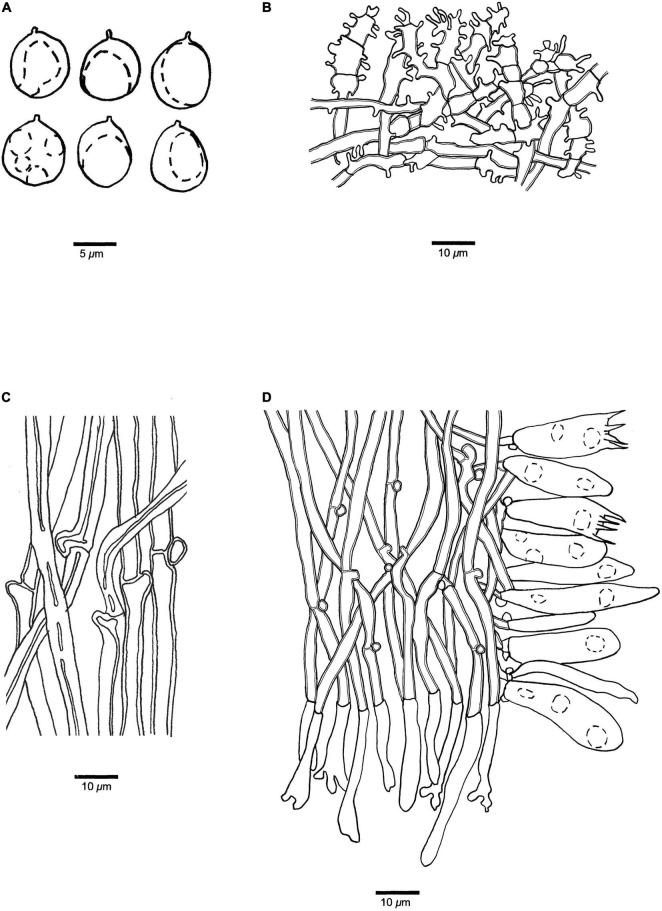
Microscopic structures of *Panellus palmicola* (holotype). **(A)** Basidiospores. **(B)** Dichophysial hyphae from pileipellis. **(C)** Hyphae from stipe. **(D)** A section of tube trama, including pleurocystidia, cheilocystidia, basidia, and basidioles. The dotted circles represent some guttules in basidiospores, basidia or basidioles.

***Panellus palmicola*** Q. Y. Zhang, F. Wu, and Y. C. Dai, sp. nov., Figures 3E, 4D, 9.

MycoBank: MB844368.

**Type.** Guangdong Province, Guangzhou, Baiyunshan, on *Trachycarpus fortune*, 11 June 2019, Dai 19717 (holotype, BJFC 031392).

**Etymology.** *Palmicola* (Lat.): referring to the species growth on palm (*Trachycarpus fortune*).

**Basidiocarps** annual, gregarious, soft corky when fresh, and chalky when dry. **Pileus** 3–6 mm × 1–3 mm, reniform, flabelliform or ellipsoid; pileal surface cream, pale buff or cinnamon when fresh and dry, opaque, convex to plane, pruinose; margin incurved or straight, entire; context thin. **Hymenophore** concolorous with pileal surface, poroid, about 40–130 pores per basidiocarp; mature pores 2–4 per mm, round; tubes up to 0.4 mm long. **Stipe** 1.5–3 mm × 0.5–1 mm, concolorous with pileal surface, laterally attached, subcylindrical, equal in most of the width but with a slightly swollen base, pruinose on the surface.

**Basidiospores** 7–9.5(–9.8) μm × (6–)6.2–8.2(–8.5) μm, *L* = 8.33 μm, *W* = 7.16 μm, *Q* = 1.15–1.20 (*n* = 90/3), subglobose to globose, hyaline, thin-walled, smooth, with some guttules, faintly IKI+, CB–. **Basidia** 28–34 μm × 8–11 μm, clavate with some guttules, 4–spored, sterigmata 3–5 μm long; basidioles 26–32 μm × 6–8 μm, clavate or acerose. **Pleurocystidia** 20–30 μm × 3–5 μm, present in hymenium, fusiform or tubular, thin-walled. **Cheilocystidia** 22–40 μm × 3–4.5 μm, present at dissepiment edge, cylindrical or tubular, thin-walled, some with diverticulate projections at the apex. **Pileipellis** comprises abundant, dense dichophysial hyphae; dichophysial hyphae slightly thick-walled, 2–7 μm in diameter; pileocystidia absent. **Tramal** hyphae subparallel along tubes, slightly thick-walled, 3–5 μm in diameter. **Hyphae in stipe** subparallel along stipe, thick-walled, some part swollen, 3–8 μm in diameter. Clamp connections present.

Additional specimens (paratypes) examined: China, Fujian Province, Fuzhou, Fuzhou National Forest Park, living *Rhapis excelsa*, 4 June 2021, Dai 22329 (BJFC 036917), Dai 22330 (BJFC 036918), Dai 22331 (BJFC 036919), Dai 22332 (BJFC 036920), Dai 22333 (BJFC 036921), Dai 22334 (BJFC 036922), Dai 22335 (BJFC 036923); Guangdong Province, Guangzhou, Baiyunshan, on *Trachycarpus fortune*, 11 June 2019, Dai 19718 (BJFC 031393), Dai 19719 (BJFC 031394); Zhaoqing, Fengkai County, Heishiding Nature Reserve, angiosperm wood, 7 June 2019, Dai 19707 (BJFC 031382).

*Note*. *Panellus palmicola* was found in Fujian and Guangdong, subtropical China, and it grows on both monocotyledonous and dicotyledonous plants in parks.

## Discussion

In this study, 15 previously accepted poroid species of *Panellus*, *viz*. *Panellus albifavolus* Corner, *P. bambusicola*, *Panellus bambusifavolus* Corner, *Panellus brunneifavolus* Corner, *Panellus hispidifavolus* Corner, *Panellus luminescens* (Corner) Corner, *Panellus luxfilamentus* A. L. C. Chew and Desjardin, *Panellus megalosporus* Corner, *Panellus microsporus* Corner, *Panellus minimus* (Jungh.) P. R. Johnst. and Moncalvo, *Panellus orientalis* (Kobayasi) Corner, *Panellus pauciporus* Corner, *P. pusillus*, *Panellus sublamelliformis* Corner, and *P. yunnanensis*, were identified mostly based on morphological examination. Among them, only four species, viz. *Panellus bambusicola*, *P. minimus*, *P. pusillus*, and *P. yunnanensis*, were confirmed by molecular evidence. Our present study demonstrates five new poroid species of *Panellus* based on both morphological characteristics and molecular phylogenetic analyses.

*Panellus alpinus* was found in Tibet, China, at an altitude of 3,000 m. Phylogenetically, samples of *P. alpinus* (Dai 23597 and Dai 23601) formed an independent linage and were related to *P. pusillus*. Morphologically, *P. alpinus*, *P. hispidifavolus*, *P. luxfilamentus*, and *P. pusillus* share chalky basidiocarps when dry and similar basidiospores (oblong ellipsoid to cylindrical, less than 6 μm in length). However, *P. hispidifavolus* differs from *P. alpinus* by the absence of pleurocystidia in hymenium and growth on the dead leaf of *Tristania* (Corner, 1986). *Panellus luxfilamentus* is distinguished from *P. alpinus* by its larger pores (hexagonal, 3 per mm vs. round, 4–6 per mm) and the absence of pleurocystidia in hymenium (Chew et al., 2015).

There are several synonyms for *Panellus pusillus* in literature (Berkeley, 1839, 1856; Berkeley and Curtis, 1868; Singer, 1953; Corner, 1954; Burdsall and Miller, 1975). Among them, *Dictyopanus copelandii* Pat. [≡*Panellus copelandii* (Pat.) Burds. and O. K. Mill.] was described from Philippines (Patouillard, 1914) and *Dictyopanus gloeocystidiatu*s Corner [≡ *Panellus gloeocystidiatus* (Corner) Corner] was described from Japan (Corner, 1954). *Dictyopanus copelandii* differs from *Panellus alpinus* by its longer stipe (4–6 mm vs. 0.4–1 mm in length) and larger basidiospores (6.5–9 μm × 3.5–5.5 μm vs. 4.8–6 μm × 2.8–3.6 μm, Burdsall and Miller, 1975). *Dictyopanus gloeocystidiatus* is distinguished from *Panellus alpinus* by its smaller basidiospores (3.2–4 μm × 2–2.3 μm vs. 4.8–6 μm × 2.8–3.6 μm) and thinner basidia (15–19 μm × 3.5–4 μm vs. 17–24 μm × 4–6 μm, Corner, 1954).

Phylogenetically, *Panellus minutissimus* is closely related to *P. bambusicola* and *P. yunnanensis* (Figures 1, 2). However, *P. bambusicola* differs from *P. minutissimus* by smaller basidia (15–18 μm × 5–6.5 μm vs. 18–21 μm × 6–8 μm) and the absence of pleurocystidia in hymenium (Zhang and Dai, 2021). *Panellus yunnanensis* is distinguished from *P. minutissimus* by its reniform and shell-shaped basidiocarps, longer basidia (20–28 μm × 5–8 μm vs. 18–21 μm × 6–8 μm), and smaller cheilocystidia (10–20 μm × 7–10 μm vs. 20–24 μm × 10–12 μm; Zhang and Dai, 2021). Morphologically, *P. albifavolus* and *P. minimus* are similar to *P. minutissimus* by sharing gelatinous basidiocarps, growth on palm (*Trachycarpus fortune*), bamboo (Bambusoideae), and other monocotyledons. However, *P. albifavolus* is distinguished from *P. minutissimus* by its larger basidiocarps (up to 3 mm vs. 0.2–0.6 mm), wider basidiospores (7–8.5 μm × 4.7–6 μm vs. 6–8 μm × 3.2–4.2 μm), and the absence of pileocystidia (Corner, 1986). *Panellus minimus* differs from *P. minutissimus* by its larger basidiocarps (0.9–3.5 mm vs. 0.2–0.6 mm) and long stipe (up to 2 mm; Johnston et al., 2006).

In both nrITS and nrITS + nrLSU + mtSSU + nrSSU + *tef*1 based phylogenies (Figures 1, 2), samples of *Panellus crassiporus*, *P. longistipitatus*, and *P. palmicola* formed three distinct lineages nested in a subclade, and they are closely related. Morphologically, species in the subclade are characterized by chalky basidiocarps when dry and similar basidiospores (subglobose to globose, more than 6 μm in width). However, *P. longistipitatus* is distinguished from *P. crassiporus* by its longer stipe (1–3 mm vs. 0.1–1.3 mm in length) and the presence of pileocystidia. *Panellus palmicola* differs from *P. crassiporus* in having larger pores (2–4 per mm vs. 4–6 per mm) and longer stipe (1.5–3 mm vs. 0.1–1.3 mm in length). *Panellus palmicola* differs from *P. longistipitatus* by its larger pores (2–4 per mm vs. 4–6 per mm), smaller pleurocystidia (20–30 μm × 3–5 μm vs. 35–53 μm × 3–6 μm), and the absence of pileocystidia in hymenium. Furthermore, *P. brunneifavolus*, *P. megalosporus*, and *P. orientalis* have globose basidiospores (Kobayasi, 1963; Corner, 1986). However, *P. brunneifavolus* and *P. megalosporus* have big basidiospores measuring 9–12.5 μm × 8–11 μm and 13–18.5 μm × 12–16 μm, respectively, while the basidiospores are 7–9.8 μm × 5–8.2 μm in the three new species. *Panellus orientalis* is distinguished from *P. crassiporus*, *P. longistipitatus*, and *P. palmicola* by its larger basidiocarps (up to 17 mm vs. 1–6 mm in the three new species) and longer cheilocystidia (40–190 μm × 3–6 μm vs. 15–40 μm × 3–11 μm in the three new species).

Previously, *Panellus* was characterized by allantoid to narrowly elliptical, amyloid basidiospores, thick-walled tramal hyphae, the presence of cheilocystidia, and lignicolous habitat (Burdsall and Miller, 1975). However, the diversity of poroid *Panellus* was underestimated. The definition of *Panellus* has been improved with the discovery of poroid species with globose basidiospores (Kobayasi, 1963; Corner, 1986). Our investigations on the Chinese *Panellus* show that most species have globose basidiospores. In addition, most *Panellus* species were found in lowland areas or areas at an elevation below 2,000 m (Corner, 1986; Chang, 1996), while our new species *P. alpinus* was found in the mountainous area and at an elevation up to 3,000 m. Accordingly, we presume that more new poroid species of *Panellus* exist in the world, and more species will be confirmed after morphological and molecular analyses.

The main morphological characteristics of 20 poroid species of *Panellus* are listed in Supplementary Table 1, and an identification key is provided as follows:

1. Basidiospores mostly >6 μm in width, subglobose to globose……………………………………………………………………………………..2

1. Basidiospores mostly <6 μm in width, ellipsoid to cylindrical………………………………………………………………………………….7

2. Pileus up to 17 mm in the largest dimension………………………………………………………………………………0.3

2. Pileus up to 6 mm in the largest dimension………………………………………………………………………………0.4

3. Basidiospores 12–16 μm in width…………………………………………………………………..*P. megalosporus*

3. Basidiospores 6–8.5 μm in width…………………………………………………………………………*P. orientalis*

4. Basidiospores mostly >9 μm in width; pleurocystidia absent……………………………………………………………….*P. brunneifavolus*

4. Basidiospores mostly <9 μm in width; pleurocystidia present……………………………………………………………………………………0.5

5. Pores 2–4 per mm……………………………………………*P. palmicola*

5. Pores 4–6 per mm………………………………………………………….0.6

6. Stipe up to 3 mm in length; cheilocystidia pyriform……………………………………………………………*P. longistipitatus*

6. Stipe up to 1.3 mm in length; cheilocystidia tubular or narrowly clavate……………………………………………………………………*P. crassiporus*

7. Basidiospores mostly >6 μm in length……………………………………………………………………………………0.8

7. Basidiospores mostly <6 μm in length……………………………………………………………………………………16

8. Basidiocarps subgelatinous to gelatinous when dry……………………………………………………………………………………0.9

8. Basidiocarps chalky when dry……………………………………………………………………………………0.14

9. Pileus up to 3 mm in the largest dimension……………………………………………………………………………0.10

9. Pileus up to 1.5 mm in the largest dimension……………………………………………………………………………0.12

10. Stipe absent…………………………………………………………………..*P. albifavolus*

10. Stipe present……………………………………………………………….0.11

11. Pileus spherical to elliptical; pores 3–5 per mm…………………………………………………………………………..*P. minimus*

11. Pileus reniform; pores 5–7 per mm…………………………………………………………………*P. bambusifavolus*

12. Stipe present……………………………………………..*P. bambusicola*

12. Stipe absent………………………………………………………………..13

13. Pores 6–7 per mm; pleurocystidia absent…………………………………………………………………*P. yunnanensis*

13. Pores 8–10 per mm; pleurocystidia present……………………………………………………………….*P. minutissimus*

14. Basidiospores mostly >9 μm in length…………………………………………………………………..*P. luminescens*

14. Basidiospores mostly <9 μm in length……………………………………………………………………………………15

15. Pores 10 per mm; cheilocystidia absent……………………………………………………………………*P. pauciporus*

15. Pores 6 per mm; cheilocystidia present…………………………………………………………..*P. sublamelliformis*

16. Basidiospores mostly <1 μm in width…………………………………………………………………….*P. microsporus*

16. Basidiospores mostly >1 μm in width……………………………………………………………………………………..0.17

17. Pleurocystidia and pileocystidia present*P. alpinus*

17. Pleurocystidia or pileocystidia absent……………………………………………………………………………………0.18

18. Pores 5–7 per mm………………………………………*P. hispidifavolus*

18. Pores <5 per mm…………………………………………………19

19. Pileus 2.5–5 mm……………………………………….*P. luxfilamentus*

19. Pileus 5–20 mm………………………………………………..*P. pusillus*

## Data Availability Statement

The datasets presented in this study can be found in online repositories. The names of the repository/repositories and accession number(s) can be found in the article/Supplementary Material.

## Author Contributions

Q-YZ, FW, and Y-CD designed the research and contributed to data analysis and interpretation. Q-YZ and MZ prepared the samples and drafted the manuscript. VP discussed the results and edited the manuscript. All authors contributed to the article and approved the submitted version.

## Conflict of Interest

The authors declare that the research was conducted in the absence of any commercial or financial relationships that could be construed as a potential conflict of interest. The handling editor declared a past collaboration with one of the author VP.

## Publisher’s Note

All claims expressed in this article are solely those of the authors and do not necessarily represent those of their affiliated organizations, or those of the publisher, the editors and the reviewers. Any product that may be evaluated in this article, or claim that may be made by its manufacturer, is not guaranteed or endorsed by the publisher.
