# Supplementary material for: Taxonomy and Multi-Gene Phylogeny of Poroid Panellus (Mycenaceae, Agaricales) With the Description of Five New Species From China
Source: Front Microbiol. 2022 Jul 27;13:928941. doi: 10.3389/fmicb.2022.928941 (PMC9363832; doi:10.3389/fmicb.2022.928941)
Supplement: Supplementary file 1 [file Table_1.DOCX]

Supplementary Material

Table 2. The main characteristics of poroid species of *Panellus*

| Species | Type locality | Basidiocarps texture (when dry) | Pileus size (mm) and shape | Pilei color | Pores (per mm) | Stipe | Basidiospores size (μm) and shape | Pleurocystidia size (μm) and shape | Cheilocystidia size (μm) and shape | Pileocystidia size (μm) and shape | references |
| --- | --- | --- | --- | --- | --- | --- | --- | --- | --- | --- | --- |
| *P.* *albifavolus* | Malaysia | gelatinous | up to 3, reniform | white | 5–6 | – | 7–8.5 × 4.7–6, broadly ellipsoid | absent | 15–30 long | 18–38 × 3–10, with diverticulate projections at the apex | Corner (1986) |
| *P. alpinus* | China | chalky | 1.5–4, flabelliform, semicircular or ellipsoid | white to cream | 4–6 | present | 4.8–6 × 2.8–3.6, oblong ellipsoid | 20–36 × 3–4, narrowly clavate and apiculate | 25–45 × 2–5, subulate to long fusiform, some with diverticulate projections at the apex | 22–47 × 3–5, narrowly clavate, some with diverticulate projections | present study |
| *P. bambusicola* | China | gelatinous | 0.5–1, spherical to elliptical | pure white to white when fresh, pale cream to ivory when dry | 8–10 | present | 6–7.2 × 3–3.8, oblong ellipsoid | absent | 22–27 × 6–8, long clavate to barrel-shaped | absent | Zhang and Dai (2021) |
| *P. bambusifavolus* | Solomon Islands | a thin gelatinous pellicle | up to 3, reniform | pale fawn | 5–7 | present | 7–9 × 3.5–4.5, subcylindrical | absent | 3–5 μm wide, cylindrical | 0–85 × 3–5, cylindrical | Corner (1986) |
| *P. brunneifavolus* | Solomon Islands | subgelatinous, with chalky edges | up to 3, reniform | white then pale fawn brown | 5–7 | absent | 9–12.5 × 8–11, subglobose or ovoid | absent | 18–45 × 3–4.5, subcylindrical | subcylindrical | Corner (1986) |
| *P. crassiporus* | China | chalky | 2–5, reniform or flabelliform | white to pale buff | 4–6 | present | 8–9.8 × 6.9–8, subglobose to globose | 18–38 × 3–7, tubular | 22–30 × 3–5, tubular or narrowly clavate | absent | present study |
| *P. hispidifavolus* | Malaysia | chalky | up to 3, reniform | white | 5–7 | present | 4.5–6× 2.5–3.3, ellipsoid | absent | like encrusted spinulose hyphal ends | absent | Corner (1986) |
| *P. longistipitatus* | China | chalky | 1–4.2, reniform to semicircular | white to greyish yellow | 4–6 | present | 7–9.8 × 5–7, broadly ellipsoid to subglobose | 35–53 × 3–6, tubular with tapered at the apex | 15–28 × 7–11, pyriform | 17–40 × 5–8, narrowly clavate | present study |
| *P.* *luminescens* | Malaysia | chalky | 2–4, reniform or flabelliform | white to pale alutaceous or pale buff | 4–8 | present | 9–13 × 5–6, elongate pip-shaped to oblong ellipsoid | absent | 30–55 × 7–10, narrowly clavate | narrowly clavate with intricately diverticulate at the apex | Corner (1986) |
| *P. luxfilamentus* | Malaysia | chalky | 2.5–5, flabelliform | greyish yellow | 3 | present | 3.6–4.4 ×2.4–2.8, ellipsoid to elongate | absent | 16–34.4 × 2.4–4, cylindrical to irregular | absent | Chew *et al.* (2015) |
| *P. megalosporus* | Malaysia | chalky | up to 22, reniform | white | 0.8–5 | absent | 13–18.5 × 12–16, subglobose or broadly ellipsoid | absent | present | absent | Corner (1986) |
| *P. microsporus* | Malaysia | chalky | up to 4, pendent, dorsifixed, discoid | fawn brown | 10–14 | present | 3–4 × 0.8, allantoid | absent | 25–80 × 7–12, clavate to subcylindrical, with diverticulate at the apex or distal part | present, similar to the cheilocystidia | Corner (1986) |
| *P. minimus* | Java | gelatinous | 0.9–3.5, spherical to elliptical | white | 3–5 | present | 6–8.5 × 3.5–5, ellipsoid to broadly ellipsoid | absent | broad-cylindrical to clavate | – | Johnston *et al.* (2006) |
| *P. minutissimus* | China | gelatinous | 0.2–0.6, conchoid, semicircular or ellipsoid | pure white to white when fresh, pale cream to ivory when dry | 8–10 | absent | 6–8 × 3.2–4.2, ellipsoid | 15–25 × 2.5–4, tubular with slightly curved at the apex | 20–24 × 10–12, pyriform | 12–16 × 6–9, pyriform | present study |
| *P. orientalis* | Japan | chalky | up to 17, reniform | white, pale buff to pale fawn-ochraceous | 4–7 | present | 6.5–9.5 × 6–8.5, subglobose | absent | 40–190 × 3–6, cylindrical to subclavate | present | Corner (1986) |
| *P. palmicola* | China | chalky | 3–6, reniform, flabelliform or ellipsoid | cream, pale buff to cinnamon | 2–4 | present | 7–9.5 × 6.2–8.2, subglobose to globose | 20–30 × 3–5, fusiform or tubular | 22–40 × 3–4.5, cylindrical or tubular | absent | present study |
| *P. pauciporus* | Solomon Islands | chalky | up to 1.5 | white | 10 | absent | 6–7.7 × 3–4, subcylindrical | absent | absent | absent | Corner (1986) |
| *P.* *pusillus* | Hispaniola | chalky | 5–20, reniform to semicircular | white to pale | 4–5, elongated radially 2–3 | **–** | 4–5.5 × 2–3, ovoid to broadly ovoid | 20–35 × 3–4, cylindrical to lanceolate | cylindrical | absent | Burdsall and Miller (1975) |
| *P. sublamelliformis* | Solomon Islands | chalky | up to 3, reniform | white then pale fawn brown, pinkish when old | 6 | absent | 6–7.5 × 4.5–5.5, broadly ellipsoid | absent | 22–35 × 7–18, clavate | present | Corner (1986) |
| *P. yunnanensis* | China | gelatinous | 0.5–1.5, reniform and shell-shaped | pure white to white when fresh, pale cream to ivory when dry | 6–7 | absent | 6.5–8.5 × 3.8–4.5, ellipsoid to broadly ellipsoid | absent | 10–20 × 7–10, clavate to pyriform | absent | Zhang and Dai (2021) |
